# Sorghum mutant *RG* displays antithetic leaf shoot lignin accumulation resulting in improved stem saccharification properties

**DOI:** 10.1186/1754-6834-6-146

**Published:** 2013-10-09

**Authors:** Carloalberto Petti, Anne E Harman-Ware, Mizuki Tateno, Rekha Kushwaha, Andrew Shearer, A Bruce Downie, Mark Crocker, Seth DeBolt

**Affiliations:** 1 Plant Physiology, Department of Horticulture, Agricultural Science Center North, University of Kentucky, Lexington, KY 40546, USA; 2 Center for Applied Energy Research, University of Kentucky, 2540 Research Park Drive, Lexington, KY 40511, USA

**Keywords:** Phenylpropanoid, Sorghum, Cell wall, Lignocellulose, Biofuel, Lignin

## Abstract

**Background:**

Improving saccharification efficiency in bioenergy crop species remains an important challenge. Here, we report the characterization of a Sorghum (*Sorghum bicolor* L.) mutant, named *REDforGREEN* (*RG*), as a bioenergy feedstock.

**Results:**

It was found that *RG* displayed increased accumulation of lignin in leaves and depletion in the stems, antithetic to the trend observed in wild type. Consistent with these measurements, the *RG* leaf tissue displayed reduced saccharification efficiency whereas the stem saccharification efficiency increased relative to wild type. Reduced lignin was linked to improved saccharification in *RG* stems, but a chemical shift to greater S:G ratios in *RG* stem lignin was also observed. Similarities in cellulose content and structure by XRD-analysis support the correlation between increased saccharification properties and reduced lignin instead of changes in the cellulose composition and/or structure.

**Conclusion:**

Antithetic lignin accumulation was observed in the *RG* mutant leaf-and stem-tissue, which resulted in greater saccharification efficiency in the *RG* stem and differential thermochemical product yield in high lignin leaves. Thus, the red leaf coloration of the *RG* mutant represents a potential marker for improved conversion of stem cellulose to fermentable sugars in the C4 grass Sorghum.

## Introduction

Second generation biofuels for fossil fuel replacement [1,2] will likely involve the grasses becoming a focal crop [3-5]. For biofuels, an attractive trait among the grasses has been C4 photosynthesis, which is particular to specific clades of the Gramineae, most prominently the Panicoideae. Traits of interest include increased CO_2_ fixation efficiency, water use efficiency and drought tolerance [6,7]. Despite the many beneficial traits of C4-grasses, the conversion of cellulosic carbohydrate to liquid transportation biofuel is limited by the recalcitrant influence upon enzymatic hydrolysis of one the most abundant component of the plant cell wall, lignin [8-13]. Lignin, a complex heterologous polymer derived primarily from three hydroxycinnamil alcohol monomers with different extents of methoxylation, namely, p-coumaryl-, coniferyl- and sinapyl-alcohols, is synthesised through the phenylpropanoid pathway [14,15]. Many advances have occurred in elucidating the genes and the genetic modules involved in this complex pathway [14,16]. Regulatory mechanisms are often mediated through metabolic intermediates [17,18] suggesting a dynamic balancing act within the phenylpropanoid pathway that could be open to re-wiring.

A class of low lignin mutants that were discovered in maize (first identified in 1924 in Minnesota [19]) were the brown midrib mutants (*bm*). The *bm* mutants were named based on the red – brown coloration of the lamina midrib, which intriguingly accompanied low levels of lignification in stem tissue [20,21]. General screening of chemical mutagenesis populations subsequently expanded the number of allelic and non-allelic *brown midrib* lines in maize and sorghum [22]. For instance, allelism studies established that many of the sorghum *bmr* mutants arose from deleterious mutations in caffeic acid O-methyl transferase (*COMT*) [22,23], although evidence of sorghum *bmr* arising from cinnamyl alcohol dehydrogenase (*CAD*) were also discovered [24-26]. Notably, with exception of the brown midrib phenotype, the *bmr* mutants display little alteration in gross morphogenesis compared with WT, but as a feedstock have lower lignin content and thus greater cellulose digestibility [23,27]. However, double *bmr* mutants have been shown to display delayed development and infertility, indicating a limitation on the extent to which lignin biosynthesis can be suppressed [28]. Nonetheless, a clear relationship between lignin biosynthesis and saccharification efficiency has been highlighted using the *bmr* mutants. It remains unclear whether along with the *bmr* phenotype, other phenotypic markers exist for altered phenylpropanoid metabolism and digestibility.

Della is a sweet sorghum [*Sorghum bicolor* (L.) Moench](Reg. no.CV-130, PI566819) developed in the 1980’s by R.L Harrison (Virginia Tech University) for the Sorghum syrup production. The cultivar was derived from a cross between the Dale and ATx622′. The diploid Della variety was selected from this cross by the pedigree breeding methodology for six generations and determined to be pure breeding in 1990. The main advantage over Dale, is that Della flowers a week earlier, maintains excellent drought tolerance and disease resistance to anthracnose pathogens. Here, the dominant *REDforGREEN* (*RG*) mutant was generated through chemical mutagenesis (ethyl methanesulfonate EMS) in the Della variety. The *RG* mutant was identified through a phenotypic screen for enhanced red pigmentation in plant tissues. It is demonstrated that the *RG* mutant displays an antithetic abundance/reduction of lignin in a tissue specific manner. We report the investigation of physical and chemical properties of the cell wall in *RG* and determine the suitability of *RG* for bioconversion.

## Results

### *REDforGREEN (RG)* sorghum mutant: trait inheritance evaluation

In sweet sorghum Della [*Sorghum bicolor* (L.) Moench](Reg. no.CV-130, PI566819), the phenotype of highly pigmented leaves and low lignin stem arose from the M_1_ and segregated in subsequent M_2_ generation in a dominant manner. We found that the *RG* phenotype displayed dominant inheritance and was carried through subsequent generations. Backcrossing into parental Della over two backcrossed generations was achieved. From the segregating F_2_ populations we recovered 761 plants 567 were phenotypically *RG* whereas 194 were phenotypically wild type in a ratio near to 3:1 (χ^2^ = 0.04892 df = 1, P > 0.05), illustrating dominant inheritance, consistent with a single mutant gene. The *RG* phenotype was found to be indistinguishable from the homozygote, therefore fully penetrant in the heterozygous state. Further, when the *RG* heterozygote was backcrossed into a wild type Della plant we obtained a near 1:1 (*RG*-phenotype to wild type-phenotype) ratio consistent with a single dominant gene segregating in a mendelian manner. The wild type, heterozygous and homozygous segregants were further analyzed in the selfed F_3_ generations to ensure segregation and selection of homozygotes for second round backcrossing. In wild type, no reoccurrence of the *RG* phenotype was observed, consistent with dominant inheritance of the trait.

### *RG* mutant displays hyper-accumulation of pigments and reduced plant height

**Figure 1:**
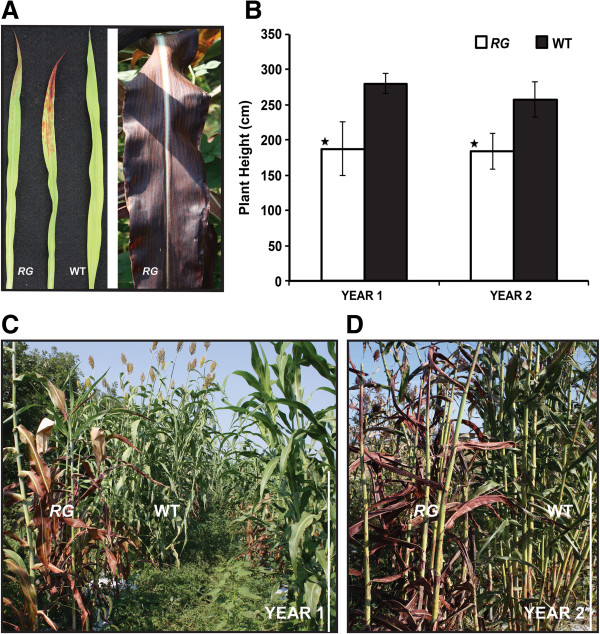
***RG *phenotype and plant height determination. (A)** Left panel, early development of the pigment accumulation phenotype; right panel a mature leaf. **(B)** Plant height of *RG* and wild type for two field seasons **(C**, **D)**. For each growing season, plant height was determined based on maximal flag termini (n = 25 replicates). Error bars represent the standard error from the mean. Scale bar, 1 m. Significance (P < 0.05) is indicated by a star (^★^).

**Figure 2:**
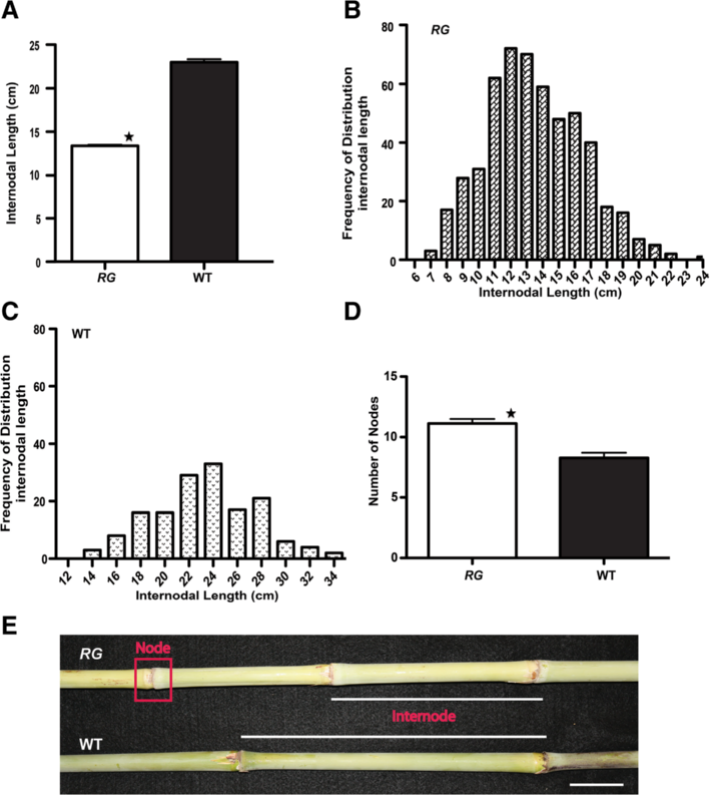
**Internode and node assessment.** The length of the internode was compared between *RG* and wild type (n = 25) **(A)**. Histograms display the distribution of internode lengths for each genotype (**B**, *RG*; **C**, WT). The average number of nodes per stems **(D)**. Error bars in all figures are standard error from the mean. Significance (P < 0.05) is indicated by a star (^★^). Visually, the node and internodes are depicted in **(E)**. Scale bar = 3.5 cm.

The *RG* mutant was phenotypically identified by a marked accumulation of red/purple pigments in the leaf blades (Figure 1A). This trait was characterized as leaf-specific and it evolved in a basipetal manner, from the tip of the leaf to the base and further to the leaf sheath (Figure 1A). The initial evaluation was completed in a temperature-controlled glasshouse and all leaves, in a progressive manner, manifested the phenotype thus resulting in a notable red/purple plant (Figure 1). To explore the mature *RG* plant phenotype outside the greenhouse, a field performance trial was established over two growing seasons. *RG* displayed a reduction in maximum height, compared with wild type. Results showed an average decrement of 37% (P < 0.0001, Mann–Whitney) during the first year and 40% (P < 0.0001, Mann–Whitney) for the second year (Figure 1B, C, D). Reduction in height could arise in association with three phenotypes: 1) reduced internodal length, 2) fewer nodes initiated during the life cycle or 3) both fewer internodes of a shorter length. A large internodal length decrease was calculated for the *RG* mutant as compared to the wild type (Figure 2A and E, P < 0.0001). Notably, the number of nodes was significantly greater in *RG* versus wild type (Figure 2D, E; P < 0.0001) but this could not compensate for the shorter internode length for *RG*, resulting in shorter plants. Additionally, the frequency distributions of internode lengths between the two genotypes indicated that the *RG* mutant is skewed toward shorter internodes relative to a more normal distribution of overall longer lengths in WT (Figure 2B and C). Leaf length and width was also estimated but was not significantly different (Data not shown). Further, it was found that the average seed weight (1000 seeds weight, with replication) was not different between *RG* and the wild type (*RG*, 1.80 ± 0.04; WT, 1.85 ± 0.03), which was consistent with no visible change in the *RG* inflorescence. Moreover, no changes in the nature and the extent of bran pigmentation, brown for Della variety, were observed.

### Cell wall composition in the *RG* mutant reveals modified lignification

**Figure 3:**
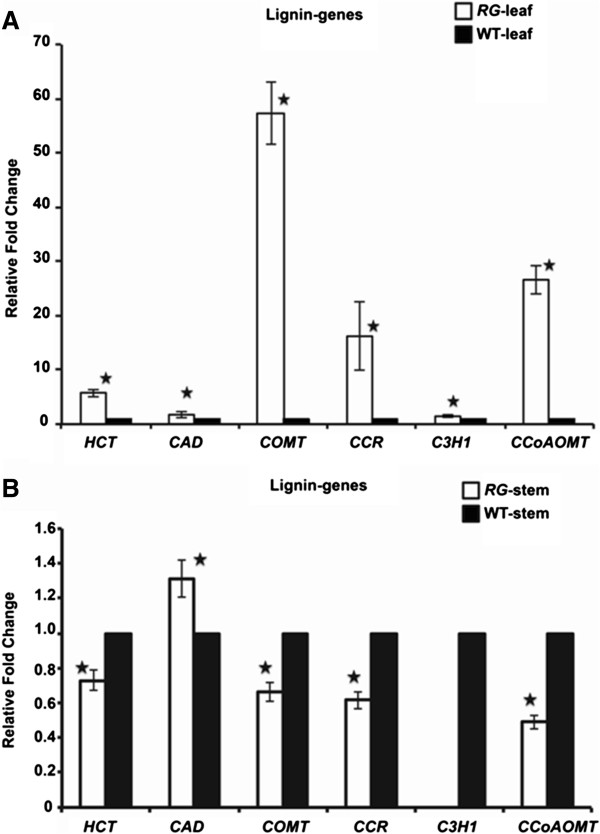
**Gene expression analysis for key genes in the lignin biosynthetic pathway. (A)** Transcriptional analysis for leaf tissues; **(B)** Transcriptional analysis for stem material at 4-week old for RG and wild type. *HCT***,***Hydroxycinnamoyl transferase; CAD*, *Cinnamyl alcohol dehydrogenase; COMT, Caffeic acid O-methyltransferase; CCoAOMT, Caffeoyl-CoA O-methyltransferase; CCR, Cinnamoyl-CoA reductase, C3H1, 4-coumaric acid 3′-hydroxylase 1.* Significance (P < 0.05) is indicated by a star (^★^).

**Figure 4:**
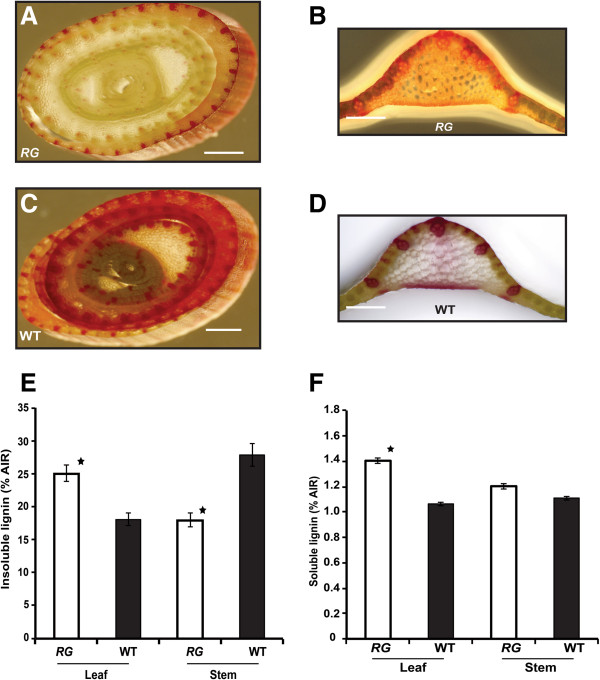
**Lignin quantification from the leaf and stem of *RG *and wild type sorghum. (A, B, C, D)** Maule’s staining for lignin in cross sections of sorghum stem **(A, C)** and leaf tissue **(B, D)**. **(E)** Total insoluble lignin, **(F)** total soluble lignin; Each bar comprises the mean of four biological and four technical replicates. Error bars indicate the standard error from the mean. Significance (P < 0.05) is indicated by a star (^★^). Scale bar = 1 mm.

As mentioned above, the *RG* mutant displays visible red coloration of leaves that develops basipetally (Figure 1A). The red/purple accumulation in *RG* leaves was consistent with an alteration in the phenylpropanoid pathway. This pathway is also responsible for the production of lignin therefore we anticipated that the genes responsible for the production of pigments and for lignin could be altered as well. To test this hypothesis, gene expression-and chemical compositional-analyses were performed. An increase in transcript abundance was observed for key genes involved in the lignin biosynthetic branch of the phenylpropanoid pathway in *RG* red leaves compared with wild type (Figure 3A). The converse was true for gene expression in the stems for all but cinnamyl alcohol dehydrogenase (Figure 3B). Consistent with these data, transcript abundance extended from those genes whose products are responsible for the earliest committed metabolic conversions in the phenylpropoanoid pathway (Petti et al., cosubmitted). To establish whether lignin biosynthesis was increased, we visually observed lignin by counterstaining with phloroglucinol stain (Maule’s reagent, [29]). Here, we examined transverse cross sections of *RG* and wild type stems and leaves. Results illustrated a more pronounced lignin staining in the wild type stem section relative to *RG* (Figure 4A,C) and *vice versa* in the leaf (Figure 4B,D). Therefore, histochemical data suggest that stems of the *RG* mutant have reduced lignin biosynthesis whereas the leaves display increased levels. Transcriptional characterization of 4-week old *RG* and WT stems demonstrated a down-regulation for key genes involved in the lignin pathway for *RG* compared to the wild type, corroborating the histochemical evidence for lignin reduction (Figure 3B). One exception was *CAD,* whose expression was up-regulated, contradictory to reduced lignification of this tissue.

The content of lignin (soluble and insoluble) in leaves and stems of field-grown *RG* and wild type plants was determined. Both forms of lignin were increased significantly in the leaf tissue of *RG* compared with wild type (Figure 4E,F). By contrast, acid insoluble lignin content decreased significantly in the stem of *RG* compared with wild type. Acid soluble lignin, which accounts for a small proportion (2-3%) of the total lignin, was unchanged in the *RG* and wild type stems. Results showed that the acid insoluble lignin content of the *RG* leaf was similar to that of the wild type stem (Figure 4E, P > 0.05). Taken together, these results demonstrate that lignin accumulated in an antithetic pattern in *RG*.

### Micro-scale saccharification of total biomass reveals *RG* mutant influences digestibility

**Figure 5:**
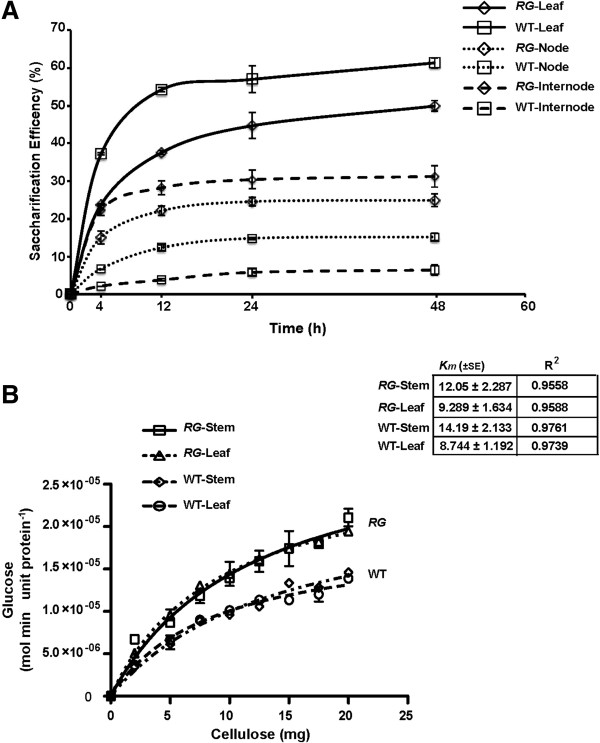
**Saccharification and kinetic properties of RG and WT biomass. (A)** Evaluation of saccharification property of *RG* and wild type tissue. Saccharification efficiency is expressed % cellulose converted to free glucose, as measured by YSI glucose analyzer (see Methods). Error bars are standard error from the mean of three biological and technical replicates. **(B)** Kinetic assessment of the digestibility of semi-purified cellulose *in vitro*. Cellulose from leaf and stem of both *RG* and wild type were evaluated by pseudo apparent Michaelis-Menten parameters to establish estimates for *K*_*m*_ and *V*_*max*_. Error bars represent the standard error from the mean of three replicates.

Based on the modified lignin content of the *RG* mutant, we sought to determine whether the lignocellulosic biomass displayed a different response to saccharification compared with wild type. It was anticipated that increased lignification in the leaves would influence saccharification efficiency. Indeed, it was found that WT leaves were more efficiently converted to fermentable sugars than *RG* leaves (Figure 5A). These data are consistent with the increased lignin content of *RG* leaves reducing digestibility. In the stems of the *RG* mutant, where lower insoluble lignin was quantified (Figure 4E), we observed that the *RG* mutant displayed higher saccharification efficiency than WT. This too supports lignin content having an influence on digestibility. Secondly, these data are consistent with the *RG* mutant phenotype being a suitable marker for digestibility traits in a tissue specific manner. As a general result, the leaves of both WT and *RG* were enzymatically deconstructed more efficiently than stems (Figure 5A).

To examine whether cellulose structure was influencing the saccharification efficiency we examined the apparent kinetic parameters of this process using semi-purified cellulose as a substrate. Enzymatic conversion of semi-purified cellulose revealed similar *V*_*max*_ for the *RG* and WT derived cellulose (Semi-purified from leaves and stems, Figure 5B). The *K*_*m*_ was determined to be very similar between *RG* (9.2 ± 1.6 mg) and WT (8.7 ± 1.2 mg).

Examination of the biomass crystallinity, which examines the crystalline signature relative to other amorphous biomass components using X-ray diffraction also showed no major difference in relative crystallinity index determinations RCI, (see Additional file 1: Figure S1). This was also supported by the analysis of semi-purified cellulose crystallinity, which also displayed no major differences (Additional file 2: Figure S2). Thus, data collectively showed that improved cellulose digestibility in the *RG* mutant stem, relative to WT, correlated best with aberrant lignification as opposed to cellulose abundance or structural changes.

### Hemicelluloses but not cellulose are altered in the *RG* mutant

**Figure 6:**
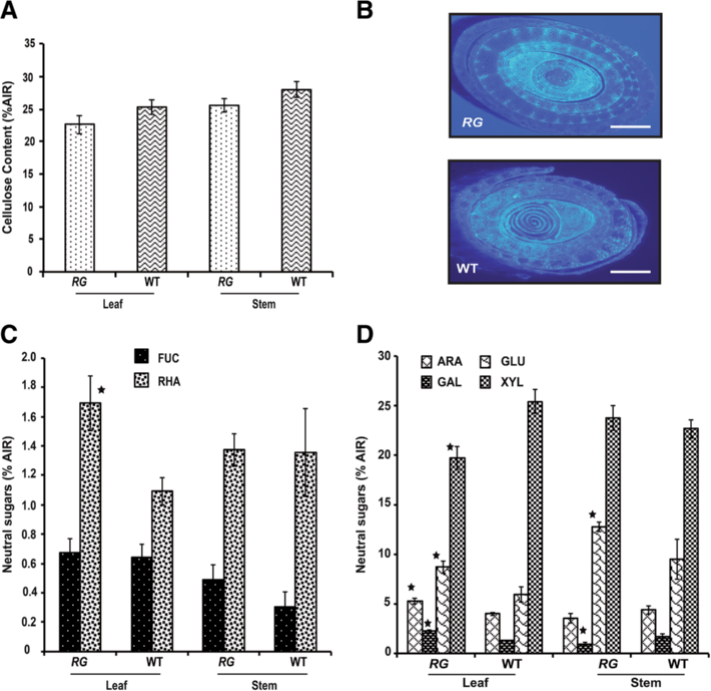
**Cellulose and hemicellulose quantification. (A)** Cellulose content from leaf and stems of *RG* and wild type sorghum (n = 4 biological and technical replicates, error bars are standard error from the mean). **B)** Histochemical analyses of cellulose deposition by Calcofluor White (Sigma Aldrich, St Louis. MO) staining of transverse sections from noted genetic background, scale bar = 1 mm. **(C)** and **(D)** Neutral sugars composition for *RG* and wild type leaf and stem biomass. (n = 4 biological and technical replicates, error bars are standard error from the mean). Significance (P < 0.05) is indicated by a star sign (^★^).

Lignin has been suggested to chemically associate with hemicellulose in the cell wall [30], particularly xylan side groups have an important role in the bonding of lignin to hemicellulose *e.g.* ester linkages between lignin and methylglucuronic acid residues and ether bonds from lignin to arabinosyl groups have been reported. Moreover phenolic lignin components, such as ferulic acid and p-coumaric acid, are covalently bound to hemicelluloses. Therefore, we hypothesized that further rearrangements in the plant cell wall may exist in the *RG* plants. To further explore, we analysed cellulose and neutral sugar composition in the leaves and stems of both genotypes. No significant difference in the percentage of cellulose was determined between the *RG* and wild type in either tissue type (Figure 6A, P > 0.05). Further, when we examined the distribution and localization of crystalline cellulose by calcofluor white staining in stems of the *RG* and wild type, we observed similar fluorescence intensity in transverse sections (Figure 6B). Neutral sugars, which contribute to the hemicellulosic fraction of the cell wall displayed variation between the *RG* mutant and wild type. Fucose and rhamnose, which in general account for small portions of the total sugars [31], were unchanged in the stems. In leaves, rhamnose was significantly greater in *RG* (Figure 6C). Also in leaves, arabinose, galactose, and glucose were significantly more abundant in *RG* than wild type (Figure 6D, P < 0.05). By contrast, leaf xylose decreased in *RG* from 26% in wild type to 19%. The stem composition also displayed differences. Here, galactose was decreased significantly and glucose was increased in *RG* (Figure 6D, P < 0.05). Therefore, neutral cell wall polysaccharides were aberrant in *RG* accompanying antithetic changes in lignification, consistent with association between the fractions.

### Changes in the *RG* lignin structure

Increased lignin production in the *RG* mutant is also evidenced by its thermochemical deconstruction products. Pyrolysates originating from the lignin fraction within the biomass include phenol, guaiacol, syringol and related aromatic hydrocarbons. The relative abundance of the lignin-based pyrolysates compared to holocellulose-based pyrolysates is influenced by the relative abundance of each of these polymers within the biomass. Py-GC/MS analysis of *RG* and wild type sorghum leaves and stems generate pyrograms for qualitative and semi-quantitative analysis (Additional file 3: Table S1 and Additional file 4: Table S2, while pyrograms are shown in Additional file 5: Figure S3 and Additional file 6: Figure S4). As expected, the total area percentage of pyrolysates originating from the lignin fraction of wild type biomass was higher in the stems than in the leaves. The wild type stems also produced more pyrolysates originating from the sinapyl monomer within the lignin polymer. Hence, the S:G ratio of the stems was higher than that of the leaves. Additionally, pyrolysis of the stems produced larger amounts of 4-vinylphenol; this is most likely the result of higher coumaryl-lignin content within the stems. The pyrograms show how the relative heights and areas of the peaks from the holocellulose (retention time typically < 24 min) are lower than those from the lignin (retention time >24 min) for the stem materials in comparison with the leaves. Compared to wild type, Py-GC/MS analysis of *RG* leaves and stems (Additional file 4: Table S2 and Additional file 6: Figure S4) demonstrated that *RG* leaves produce a higher total amount of lignin-based volatile pyrolysates than the wild type leaves, which was consistent with lignin content determination. Moreover, *RG*-leaves produced more sinapyl-derived pyrolysates relative to coniferyl-derived pyrolysates than the wild type leaves and hence appear to have a higher S:G ratio based on the distribution of the volatile pyrolysates. In addition, the *RG* stems have slightly higher S:G ratios than the wild type stems and show a higher distribution of lignin-based pyrolysates in comparison to the wild-type stems. While this analysis contradicts the total lignin content determination, it may reflect the differences between the *RG* and wild type stems in the preferential formation of char from certain biopolymers. For example, the char production upon pyrolysis of the sorghum samples may differ and may incorporate varying degrees of char and nonvolatile compounds from the lignin or holocellulosic fractions. Hence, Py-GC/MS analysis may not always agree with lignin content determination. It does, however, provide information regarding the production of certain renewable bio-chemicals produced by thermal decomposition. For example, the mutant stems produced significantly higher amounts of phenol and 4-vinylphenol (P < 0.05) than the wild type stems. These pyrolysates are likely the result of increased coumaryl content within the lignin in the *RG* stems. The *RG* leaves also produced more vanillin upon pyrolysis. Taken together these data indicate that the composition and structure of the lignin polymers differed between *RG* and wild type, and are partly consistent with quantitative lignin determination.

### Thermochemical analysis of *RG*

**Figure 7:**
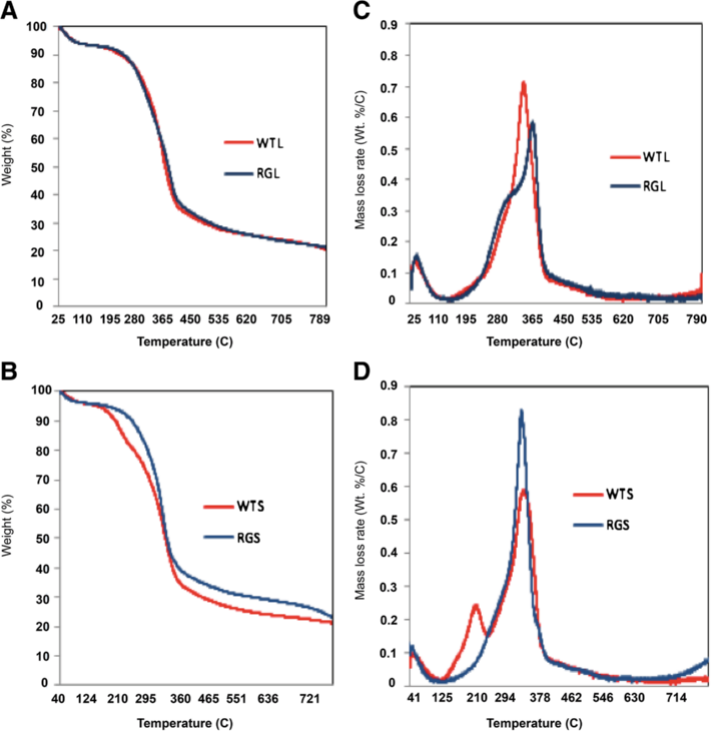
**Thermogravimetric analysis.** Weight-loss curves for leaf **(A)** and stem **(B)**. Derivative plots of thermogravimetric analysis corresponding to leaf **(C)** and stem **(D)** biomass.

Of interest was how the *RG* leaves and stems, which displayed marked changes in cell wall composition, undergo thermal decomposition in comparison with wild type. On the basis of TG curves, it is evident that the *RG* stem pyrolyzed at a higher temperature in comparison with the wild type feedstock (Figure 7B). Furthermore, it was found that the *RG* stem displayed approximately 10% less weight loss at 450°C relative to the wild type stem (Figure 7B). Neither stem nor leaf samples showed greater than 80% weight loss, which might reflect repolymerization of lignin residue forming “hard coke” [32] and it reflects the ash content present in the biomass. DTG analysis of leaves showed that *RG* biomass underwent decomposition at a higher temperature (about 365°C) compared to wild type leaves (Figure 7C) and also demonstrated a prominent shift in the main decomposition peak from 355 to 365°C. It has been reported that hemicellulose and cellulose show DTG peaks at 268°C and 355°C, respectively [33]. Wild type leaf tissue showed a single decomposition peak at 355°C, which was consistent with a strong cellulose peak. *RG* leaf decomposition took place at two different temperatures (290–300°C, 365–375°C) corresponding to two distinct DTG peaks (Figure 7C). This result suggests that modifying cell wall composition in the *RG* mutant modestly increased the pyrolysis temperature of the leaf sample. In stem analyses, the DTG curves revealed a pronounced peak at 355°C for both mutant and wild type (Figure 7D). The wild type stem also displayed a nominal pyrolysis peak at 210°C that was absent in all remaining samples and was uncharacterized. Taken together, the *RG* leaves pyrolyzed over a broader temperature regimen than the stems. It is likely that a masked broad peak of low weight loss rate occurring in all of the DTG plots from approximately 200°C to 600°C corresponds to the decomposition of the lignin in the biomass [34]. The lignin in each of the samples (leaves and stems) appears to decompose at similar rates despite differences in the DTG plots (Figure 7C, D).

### Shifts in the elemental composition of *RG*

Micronutrient abundance was assessed in *RG,* as this may indicate any notable physiological reallocations associated with the *RG* mutant and secondly due to their important for optimizing biorefining strategies. Interestingly, it was found that the wild type leaf tissue exhibited greater metal abundance than observed in *RG*. The opposite trend was observed in *RG* stems, where total metal composition was 27015 ppm compared to the WT stem total of 14437 ppm, almost 50% fewer metals in the WT stem (Table 1). Out of 11 metals examined, four were essential micronutrients (B, Mn, Zn and Fe; Table 1) and three of those, B, Mn and Zn, had between 1.5 to 3-fold greater abundance in the *RG* leaf than in the wild type. A noticeable anomaly to this trend was observed for the primary macronutrient K, which was more abundant in wild type leaves versus the *RG*. The opposite K-trend was highlighted in the stem composition and here *RG* displayed more than wild type (Table 1). Calcium (Ca) was the most abundant secondary macronutrient in all samples the *RG* leaf and stem contained around 50% more Ca than wild type. Further, the secondary macronutrient Mg was also more prevalent in *RG* leaves than in wild type. The complete analysis for C, H, N and O displayed no significant (P > 0.05) differences between the *RG* and wild type (Additional file 7: Table S3).

**Table 1:** Metal composition of biomass samples

**Biomas**												
	**As**	**Al**	**B**	**Ca**	**Cu**	**Fe**	**Mg**	**Mn**	**Na**^**5**^	**Zn**	**K**	**Sum**
WTL^1^	2	77	8	8482	13	142	1423	103	53	25	22622	32950
WTS^2^	2	100	4	1099	3	189	952	27	44	10	12007	14437
*RG*L^3^	2	52	28	12459	8	129	3347	170	195	47	13269	29706
*RG*S^4^	3	51	6	2668	6	138	1036	213	61	20	22813	27015

Biomass employed: ^1^ indicates wild type leaves, ^2^ wild type stems, ^3^*RG* leaves and ^4^*RG* stems. ^5^ C4 essential nutrient.

Values are reported in ppm.

## Discussion

Here we demonstrate that the phenylpropanoid sorghum mutant *RG* displayed antithetic leaf shoot lignification, which results in improved saccharification efficiency in the *RG* stem. Specifically, *RG* leaf samples displayed approximately 30% lower efficiency and *RG* stem was approximately 2.5-fold higher saccharification efficiency than wild type. These results correlated with an irregular distribution of insoluble lignin in the abovementioned tissues and support lignin’s inhibitory role on enzymatic processes [35]. Indeed, lignin was more abundant in *RG* leaves and less abundant in stems, when compared with wild type. Thus, the sorghum *RG* phenotype identified here represents a prominent marker for redistribution of lignin (and broadly phenylpropanoid metabolism) and improved stem saccharification efficiency for conversion of lignocellulosic biomass to fermentable sugars.

Despite evidence that lignin content and composition were altered, as was the hemicellulose fraction, no data supported a change in cellulose biosynthesis in the *RG* mutant. The proportion of cellulose as expressed per gram of cell wall was unchanged between the *RG* and wild type. Further, XRD as well as pseudo-kinetics of semi-purified cellulose saccharification failed to support a structural alteration of the cellulose microfibrils. Therefore, it seems most plausible that the alterations in saccharification arose as a consequence of the abundance and composition of lignin in the cell wall. Ancillary phenolic components such as flavonoids were also more abundant in the *RG* mutant (unpublished data). It is therefore foreseeable that lignin, flavonoids and other phenolic compounds collectively contributed to lower saccharification efficiency, consistent with prior reports [36]. Nonetheless, prior evidence supports lignin content having the most pronounced effect on saccharification efficiency [35,37-39].

The *RG* phenotype included an overaccumulation of phenolic constituents, including lignin in leaf material. The leaf tissue of the plant is less suitable for saccharification, but has several interesting properties for exploitation in a bio-based economy. Specifically, thermochemical pyrolysis coupled to GC/MS demonstrated that the increased lignin and phenolic components of the *RG* leaf tissue released a differential spectrum of high value pyrolysates including vanillin, a structural compound related to vanillin, namely the 4-hydroxy-3-methoxyacetophenone (Apocynin) and 4-hydroxybenzaldehyde; these compounds have been investigated for their pharmaceutical potentials and effect on human health [40,41]. The molecular makeup of pyrolysate (bio-oil) depends partially on the composition of the feedstock. In the case of the high lignin leaf material in the *RG* mutant, oxygenates resulting from the oxidative deconstruction of lignin could be deoxygenated to produce hydrocarbons [32] or separated to recover high-value chemicals that are present, for example upgradable building blocks such as phenols. Tailoring biomass composition as was performed herein needs to be accompanied by deconstruction chemical engineering processes to improve the range of fuels and chemicals derived. In this context, with around 13% of crude fossil oil used for the production of chemicals [42] that are important for everyday life, the identification of biomass feedstocks with the appropriate composition to replace these products needs further consideration.

*RG* leaves produced greater sinapyl-derived pyrolysates relative to coniferyl-derived pyrolysates than did WT leaves and hence appear to have a higher S:G ratio. Changes in S:G ratio can influence degradation properties, in terms of hydrolytic enzymes creating fermentable sugars from lignocellulosic feedstocks [43]. Thus, the metabolic re-writing evident in *RG* may represent a strategy to modulate lignin structure. Lignin and other phenolic plant metabolites have typically been viewed as a waste stream in a biorefineries, which instead focus on polysaccharides as feedstock inputs [44]. In the case of the *RG* mutant, the leaf biomass would need to be removed and separated from stem material either during or post-harvest in order to optimize biorefining costs. Moreover, *RG* leaf tissues also displayed increased micronutrients. Coupled with increased phenolics such as anthocyanins, the *RG* leaf tissue may provide a feedstock for the development of nutritional supplements for livestock [45]. Indeed, high value by-products of lignocellulosic crops developed during bioprocessing has been under-explored.

The main *RG* phenotype related to bioenergy was antithetic lignification of leaves versus stems. Reduction in stem lignification has remained a goal of biotechnology efforts to reduce the recalcitrance of lignocellulosic biomass to saccharification. However, the possible genetic mechanism for control of such a shift in the PPP are not clear. In *RG* leaves*,* lignin, anthocyanin and 3-deoxyantho-cyanidins were all upregulated. This is consistent with the entire PPP being co-regulated, not just a sub-branch of the pathway. Regulation of the PPP is complex, and numerous levels of feedback inhibition and transcriptional regulation exist. For example, intermediates of the pathway negatively regulate gene expression and thus co-regulate the accumulation of precursor or the next metabolite ([17,18] and as discussed in [46]). In the stems of *RG,* down-regulation of the lignin specific PPP genes was generally observed. One exception was observed for the *CAD* transcript*,* whose expression was up regulated, contradictory to reduced lignification of the young stem tissue. The *CAD* gene encodes a cynnamyl alchohol dehydrogenase. In most higher plants, coniferyl and sinapyl aldehydes are converted to alcohols by the CAD enzyme, but also by sinapyl alcohol dehydrogenase (SAD) for the respective aldehyde [47]. Despite lignin formation resulting from a complex network of interacting genes, we anticipated that CAD would not be upregulated. It remains unclear why this reversal of gene expression to end-product metabolite occurs, but we speculate that the timing of *CAD* expression may have either be invoked as a compensatory mechanism to general down-regulation of the pathway. While possible, it is unlikely that an alternative aldehyde substrate or concentration of coniferyl aldehyde increased. Finally, it may be possible that epigenetic modifier influenced PPP elements differentially, as was recently observed for the dominant *Unstable factor for orange1* (*Ufo1*) allele [48]. Regardless, this differential expression of *CAD* remains an obvious irregularity in our dataset. The genetic mechanism underscoring *RG* must therefore over-ride genetic, developmental and physiological regulatory networks underlying the PPP. It is thus not surprising that the *RG* displayed some similarity to previously described phenotypes associated with PPP regulation, notably the light dependence of the *Lc, B* and *R* loci*. RG* also shared similarity with the carbohydrate defective mutants, *SXD* and *TYE*, whereby inability to mobilize sucrose led to a starch accumulation and early pigmentation and leaf senescence [49-51]. It is possible that the *RG* phenotype arises as a result of a block in sucrose remobilization from leaves to major physiological sinks (stem in *RG*). Such as scenario is consistent with reduced biomass and lignin in stems, sucrose remaining in leaves being deposited as lignin as alternative carbon sink and anthocyanin accumulation due to osmotic stress/phloem swelling. Similarities to the phenotypes such as single gene dominant inheritance and morphogenic phenotypes are evident between *RG* and the maize alleles that condition premature senescence in the absence of kernel formation [52,53].

Lignin alteration in *RG* was also qualitative. A change in the S:G ratio was measured in *RG* compared with wild type. Since deposition of lignin types (H, G and S) is a spatial-temporal process [15,54], the quantitative/qualitative changes could indicate that the branches of the lignin pathways are differentially regulated leading to a differential deposition of say S versus G. It is tempting to speculate that bypassing the regulatory networks responsible for physiological and developmental control may be associated with a dominant negative mutation [55]. Similarly, a mutation in the promoter region of transcription factors has been associated with the activation of the PPP. For instance, multiple repeats within a MYB10 transcription factor in apples (*Malus domestica* L.) have been coupled with an autoregulation and a dramatic all-red phenotype [56]. However, evidences of single gene autoregulation are also available in literature for instance the case for the sorghum *bmr2* mutant and a consistent down-regulation of lignin in both tissue types [57].

Taken together, the *RG* mutant leaf biomass has potential to be implemented as a dual use crop, particularly as the EMS mutation was generated in the *S. bicolor* DELLA sweet sorghum background, noted by the abundant sucrose sequestered in the stem during development [58]. However, the *RG* mutant displayed an overall reduction in the total plant height in the mature plant to around 60-70% of wild type. Several plausible explanations exist to explain this observation. Firstly, the reduction in size was consistent with the correlation between lignin content and biomass highlighted in various studies [59,60]. Here, reduced lignin results in reduced water transport capacity and suppressed height potential. Nonetheless, in a techno-economic assessment the reduced height (biomass) may result in a loss of economic profitability for using *RG,* despite the improved saccharification efficiency. Depending on the yield of high-value bio-chemicals derived from any feedstock, the biomass processing value chain becomes unsustainable. Therefore, provided the increased phenolic structures in the *RG* leaf tissue combined with the accessible carbohydrate reserves evident low lignin sweet Sorghum stems providing an intriguing feedstock for assessment.

Estimates of the DELLA cultivar yield, linked to the parental cultivar Dale, are 18.6 tons per acre of stripped stalks ready for conversion to sweet sorghum syrup (Blitzer, AGR122). With a 30% reduction in yield around 13 tons per acre would be produced. Given the 2,5-fold increase in saccharification efficiency in the *RG* stem, the resulting glucose yield for biofuel conversion may compensate for the loss in sucrose yield.

## Conclusion

Antithetic lignin accumulation was observed in the *RG* mutant leaf and stem tissue, which resulted in greater saccharification efficiency in the *RG* stem and differential thermochemical product yield in high lignin leaves. Process tailored biomass feedstocks for bio-alcohols, hydrocarbon-based renewable fuels or biobased chemicals represent an underexplored breeding end point. Data for high lignin leaves in the *RG* mutant demonstrate the potential for creating different pyrolytic breakdown products and yields, which could have important connotations for future studies.

## Materials and methods

### Plant material

Mutant and wild type (WT) seeds were sown on soil-less media (MetroMix 360, SunGro Industries Bellevue, WA). Plants were grown and maintained in a temperature-controlled glasshouse at 24°C. Soil was maintained at field water conditions and fertilized with 3 grams of Osmocoat (The Scotts Company, Marysville, OH) integrated into the soil-less media before seeding. Plants were transplanted into the field and drip irrigated into a maurey silt loam soil-type, at the University of Kentucky Horticulture Research Farm (Emmitt Road, Lexington, KY) and maintained under plasticulture. Following establishment, the plants were grown in full sunlight for 3 months. Leaf and stem color, total plant height, internode length, total node number per plant and leaf blade length (from the ligule to the leaf tip) and width at the widest point were all acquired from these plants.

### Leaf and stem RNA extraction and Q-RT-PCR analysis

Gene expression of candidate genes within the PPP was done on red leaf as well as to stems of 4-weeks old leafless sorghum *RG* and wild type plantlets. Three biological replications were harvested for each sorghum line, *RG* and wild type. RNA extraction was done by a modified hot-phenol protocol described in [61]. DNAsing, carried out by DNAse I (Fermentas), and cDNA synthesis (AB Applied Biosystem) were completed according to the manufacturer indication. SyBr green semi-quantitative assay was then completed on the biological samples and on three technical replicates on a StepOne Real time system (AB Applied Biosystem) and the ∆∆ method [62] was used for quantifying the relative expression levels normalised against the actin gene. The genes tested and primers sequences are listed in Additional file 8: Table S4.

### Lignin content determination

Acid-soluble lignin, acid-insoluble lignin and ash were measured using the laboratory analytical protocols NREL, LAP-004 (1996) and lignin distribution was visualized by phloroglucinol staining. For lignin determination, the first internodal region of field grown plants was used as well as leaf material, and 4 biological replicates (each containing 4 technical replicates) of 300 ± 1.0 mg were employed. The localization of lignin was determined histochemically on stems of axillary secondary shoots of the main mutant/WT, stem as well as on 4–6 weeks old plantlets, using a saturated solution of phloroglucinol in 20% HCl. All chemicals were reagent grade (Sigma-Aldrich, St. Louis, MO), unless noted otherwise.

### Structural characterization of the mutant biomass by thermal decomposition

The products formed from thermal decomposition of the total biomass were analyzed via Pyrolysis-GC/MS (Py-GC/MS) and thermogravimetric analysis (TGA). Pyrolysis-GC/MS experiments were performed using a Pyroprobe Model 5200 (CDS Analytical, Inc.) connected to an Agilent 7890 GC with an Agilent 5975C MS detector. The pyroprobe was operated in trap mode under a He atmosphere. Pyrolysis was conducted at 650°C (1000°C s^-1^ heating rate) for 20 s. The valve oven and transfer lines were maintained at 325°C. The column used in the GC was a DB1701 (60 m × 0.25 mm × 0.25 μm) and the temperature program was as follows: 45°C for 3 min., ramp to 280°C at 4°C min^-1^ and hold for 10 min. The flow rate was set to 1 ml min^-1^ using He as the carrier gas. The inlet and auxiliary lines were both maintained at 300°C and the MS source was set at 70 eV. The GC-MS was calibrated for a number of phenolic compounds including phenol, 2-methoxyphenol, 2-methoxy-4-methylphenol, 2,6-dimethoxyphenol, vanillin, syringaldehyde and 2-methoxy-4-vinylphenol. Pyrolysis products were analyzed according to retention time and mass spectra data obtained from a National Institute of Standards and Technology (NIST) library. The ratio among syringyl (S) and guaiacyl (G) lignin components (S:G ratio) was determined from the distribution of lignin pyrosylates according to a published method (Harman-Ware et al. 2013). TGA was performed on a TA Discovery TGA under 25 ml min^-1^ of N_2_ at a ramp of 10°C min^-1^ to 800°C followed by a ramp of 20°C min^-1^ to 1000°C. The chemical alteration and the lignin ratio were determined using a function describing the relative abundance of the determined pyrolysates.

### Neutral sugar analysis

For neutral sugar analysis, a protocol described in [63] was followed. Ribose was used as an internal sugar standard and authentic standards, used for all sugars, identified ribose and fucose, rhamnose, arabinose, galactose, glucose, mannose, xylose, galacturonic- and glucuronic-acid. The HPLC profile used was described previously [64]. Measurements comprised four biological replicates as well as four technical replicates.

### Cellulose estimation

Cellulose content was estimated as acid soluble glucose [65]. A crude cell wall extract was prepared according to [66]. Cellulose estimation was completed on 5 mg of field grown plant material and used four biological and technical replicates.

### Micro-scale saccharification of biomass

Saccharification efficiency was measured for leaf and stem material from the *RG* mutant and wild type. Briefly, total biomass was collected from field grown samples. Samples were dried at 50°C for seven days prior to homogenization using a grinder (Arthur H Thomas Co Scientific, Philadelphia, PA, USA) equipped with a 1.0 mm sieve. A cocktail of Cellulase from *Trichoderma reesei* (Sigma) and Cellobiase from *Aspergillus niger* (Sigma, USA), equivalent to 2 Filter Paper Activity Units (FPU), was used to digest 100 mg of raw biomass (ethanol and acetone washed) in a 50 mM citrate buffer (pH 4.8) in a final volume of 2 ml at 50°C. A time course deconstruction assay was performed over a period of 48 hours (at 0, 4, 12, 24 and 48 hours) whereby 3 100 μl aliquots (without replacement) were collected for glucose analysis. Quantification used the Yellow Springs Instruments (YSI)-glucose analyzer standardized for glucose determination using YSI buffer and membranes purchased from YSI (Yellow Springs, OH, USA). Glucose release was converted into saccharification efficiency and expressed as a percentage of cellulose within the biomass convertible to glucose.

### Semi-purified cellulose digestion

Cellulase digestion and pseudo apparent kinetic analysis of semi-purified cellulose from both the *RG* mutant and wild type tissue was performed as described previously [67]. Semi-purified cellulose (2, 5, 7.5, 10, 12.5, 15, 17.5 and 20 mg) obtained from leaf and stem material from *RG* and WT was digested in the presence of equal amounts of Cellulase and Cellobiase, equivalent to 2 FPU, in a 50 mM citrate buffer (pH 4.8) in a final volume of 2 ml at 50°C for 2 hours. The enzyme mixture was heat inactivated (100°C, 3 min) prior to glucose measurement using the YSI-glucose analyzer (Yellow Springs, OH, USA)(as described above). These data were generated in triplicate for average ± standard error and the glucose values converted to mol min^-1^ unit protein^-1^ and used to determine the apparent kinetics values using the program GRAPHPAD PRISM-4 (Graphpad, La Jolla, CA, USA). The inability to exactly calculate the number of catalytic ends in the complex mixture of cell wall biomass allowed only for the calculation of a relative estimation, expressed as relative kinetics (relative *V*_*max*_ and *K*_*m*_).

### X-ray diffraction analysis

X-ray diffraction was performed as described previously [68]. Diffractograms were collected between 5° and 35° (for samples with little baseline drift), with 0.02° resolution and a 2 s exposure time interval for each step.

### Statistics

Statistical analyses employed both Microsoft Excel (Seattle, WA) and GRAPHPAD PRISM-4 (La Jolla, CA) software. Significantly deviating means between WT and *RG* were determined in both instances using a Student t-test (α = 0.05). Chi-square test was employed to evaluate the “goodness of fit” of the inheritance of the trait using PRISM4.

## Abbreviations

DTG: Differential thermogravimetry; DW: Dry weight; FW: Fresh weight; GPC: Gel permeation chromatography; Py-GC-MS: Pyrolysis gas chromatography–mass spectrometry; TG: Thermogravimetry; TGA: Thermogravimetric analysis.

## Competing interests

The authors declare that they have no competing interests.

## Authors’ contributions

CP carried out the *RG* selection, biomass composition, digestibility studies and drafted the manuscript. MT, ABD, RK and AS performed biomass compositional analysis. AEHW and MC generated the GPC, TGA and DTG curves for thermochemical deconstruction, developed the pyroprobe assays and drafted the manuscript. SD conceived the study and drafted the manuscript. All authors read and approved the final manuscript.

## Supplementary Material

Additional file 1: Figure S1 X-RAY diffraction (XRD) analyses of *RG* and wild type biomass. In A, diffractograms generated from *RG* and wild type leaf material and corresponding relative cristallinity index (RCI) values. In B, XRD of stem biomasses and associated RCI values. Click here for file

Additional file 2: Figure S2 X-RAY diffraction (XRD) analyses of *RG* and wild type semi-purified cellulose. In A, diffractograms generated from *RG* and wild type semi-purified cellulose from leaf material and corresponding relative cristallinity index (RCI) values. In B, XRD of semi-purifed cellulose from stem biomasses and associated RCI values. Click here for file

Additional file 3: Table S1 Pyro-GC/MS analysis of WT biomass [Leaf (WTL) and Stem (WTS)]. Click here for file

Additional file 4: Table S2 Pryo-GC/MS analysis of *RG* biomass [Leaf (RGL) and Stem (RGS)]. Click here for file

Additional file 5: Figure S3 Representative pyrograms of wild type stem and leaf biomass. Numbered peaks on the chromatograms correspond to the peaks reported in Additional file 3: TableS1 and they are typical products seen from pyrolysis of different biomass types. (9) Furfural; (19) phenol; (20) 2-methoxyphenol, (31) 4-vinylphenol; (32) 2-methoxy-4-vinylphenol (35) 2,6-dimethoxyphenol and (45) 4-vinylsyringol. Click here for file

Additional file 6: Figure S4 Representative pyrograms of *RG* mutant stem and leaf biomass. Numbered peaks on the chromatograms correspond to the peaks reported in Additional file 4: Table S2 and they are typical products seen from pyrolysis of different biomass types. (9) Furfural; (19) phenol; (20) 2-methoxyphenol, (31) 4-vinylphenol; (32) 2-methoxy-4-vinylphenol (35) 2,6-dimethoxyphenol and (45) 4-. Click here for file

Additional file 7: Table S3 Ultimate analysis of biomass samples. Click here for file

Additional file 8: Table S4 List of genes in the phenylpropanoid pathway investigated by RT-PCR. Click here for file
