# Supplementary figures and images for: Sorghum mutant RG displays antithetic leaf shoot lignin accumulation resulting in improved stem saccharification properties
Source: Biotechnol Biofuels. 2013 Oct 9;6:146. doi: 10.1186/1754-6834-6-146 (PMC3852544; doi:10.1186/1754-6834-6-146)

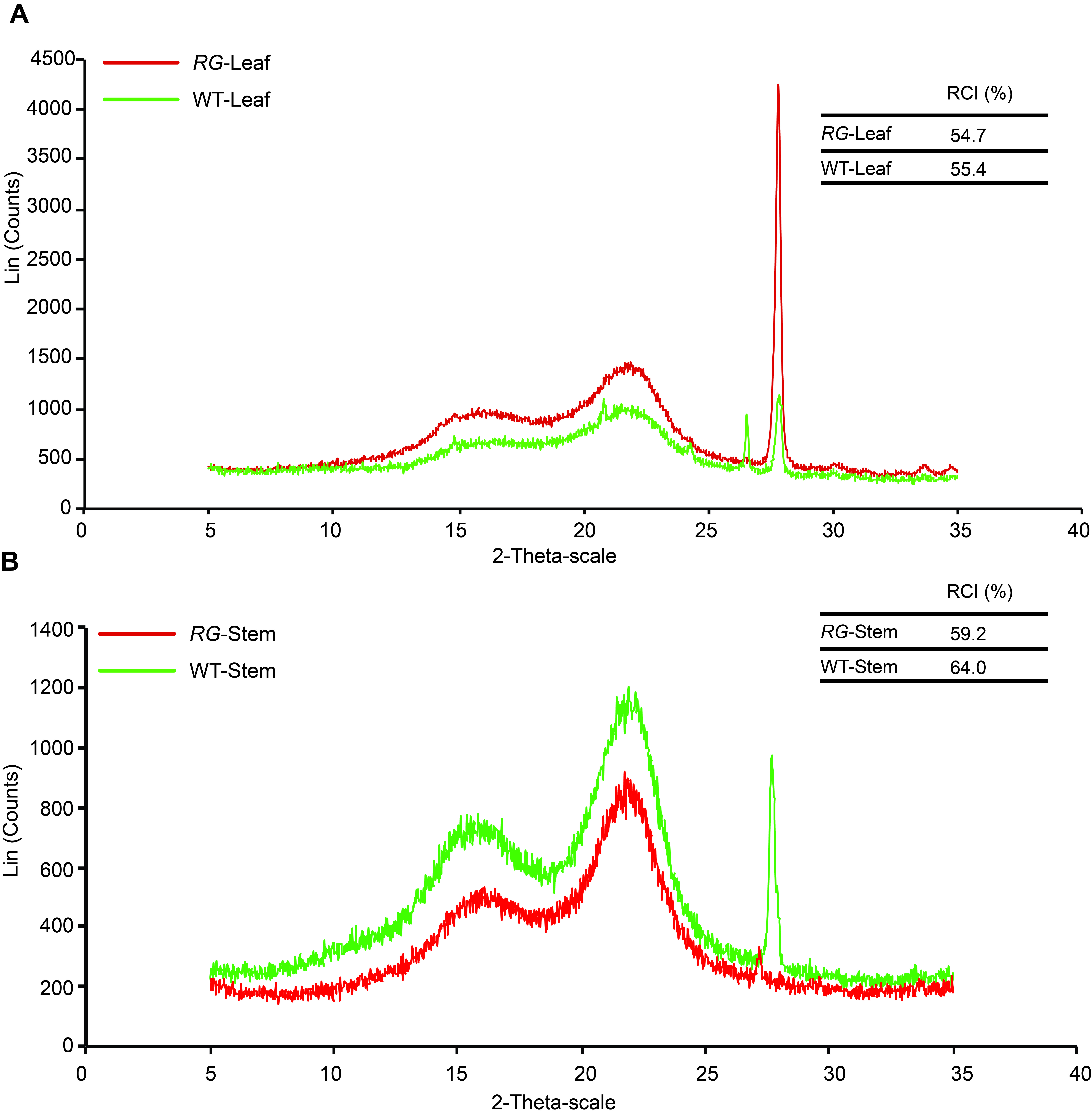

Supplement: Additional file 1: Figure S1 — X-RAY diffraction (XRD) analyses of RG and wild type biomass. In A, diffractograms generated from RG and wild type leaf material and corresponding relative cristallinity index (RCI) values. In B, XRD of stem biomasses and associated RCI values. [file 1754-6834-6-146-S1.jpeg]

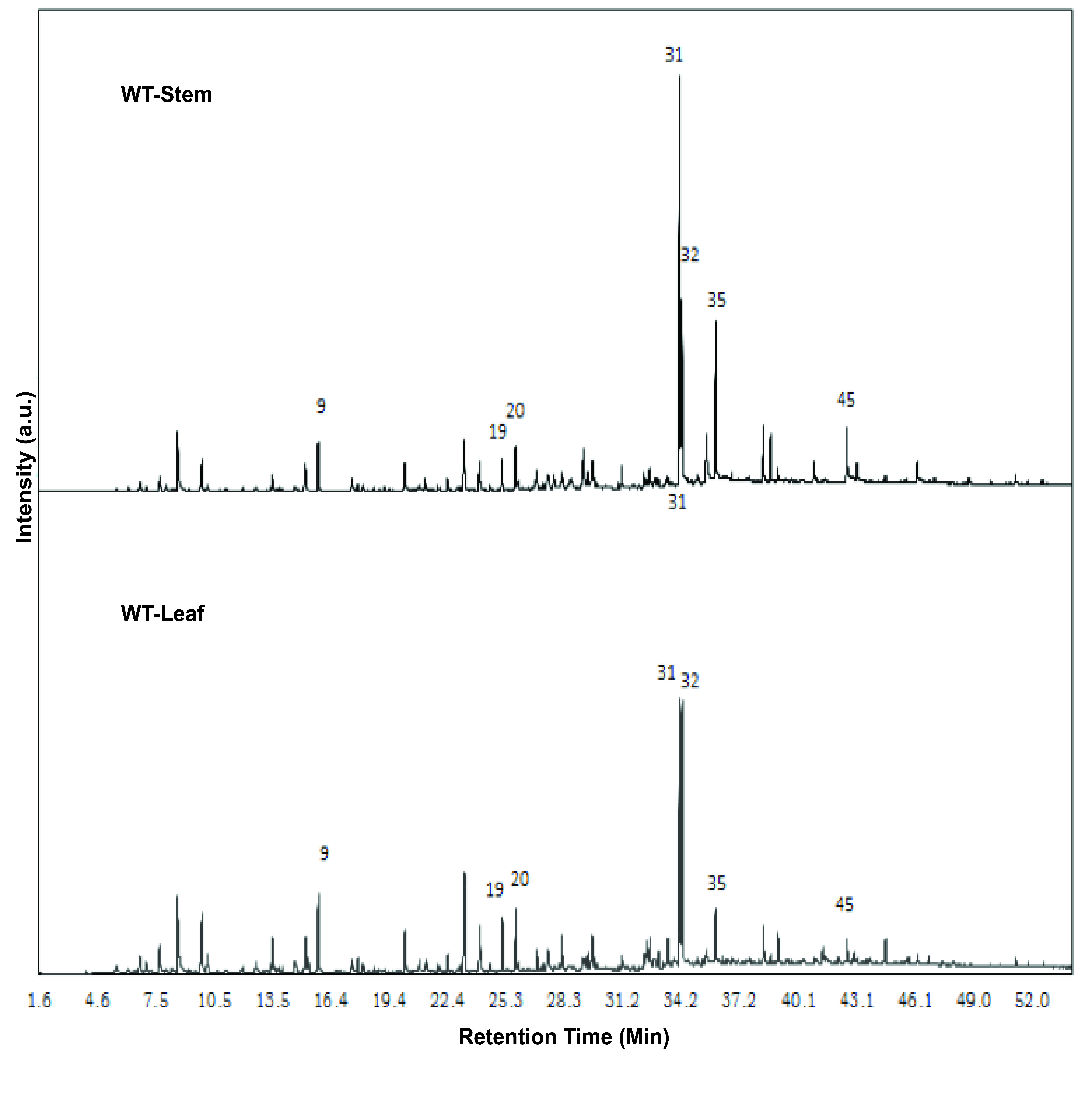

Supplement: Additional file 5: Figure S3 — Representative pyrograms of wild type stem and leaf biomass. Numbered peaks on the chromatograms correspond to the peaks reported in Additional file 3: TableS1 and they are typical products seen from pyrolysis of different biomass types. (9) Furfural; (19) phenol; (20) 2-methoxyphenol, (31) 4-vinylphenol; (32) 2-methoxy-4-vinylphenol (35) 2,6-dimethoxyphenol and (45) 4-vinylsyringol. [file 1754-6834-6-146-S5.jpeg]

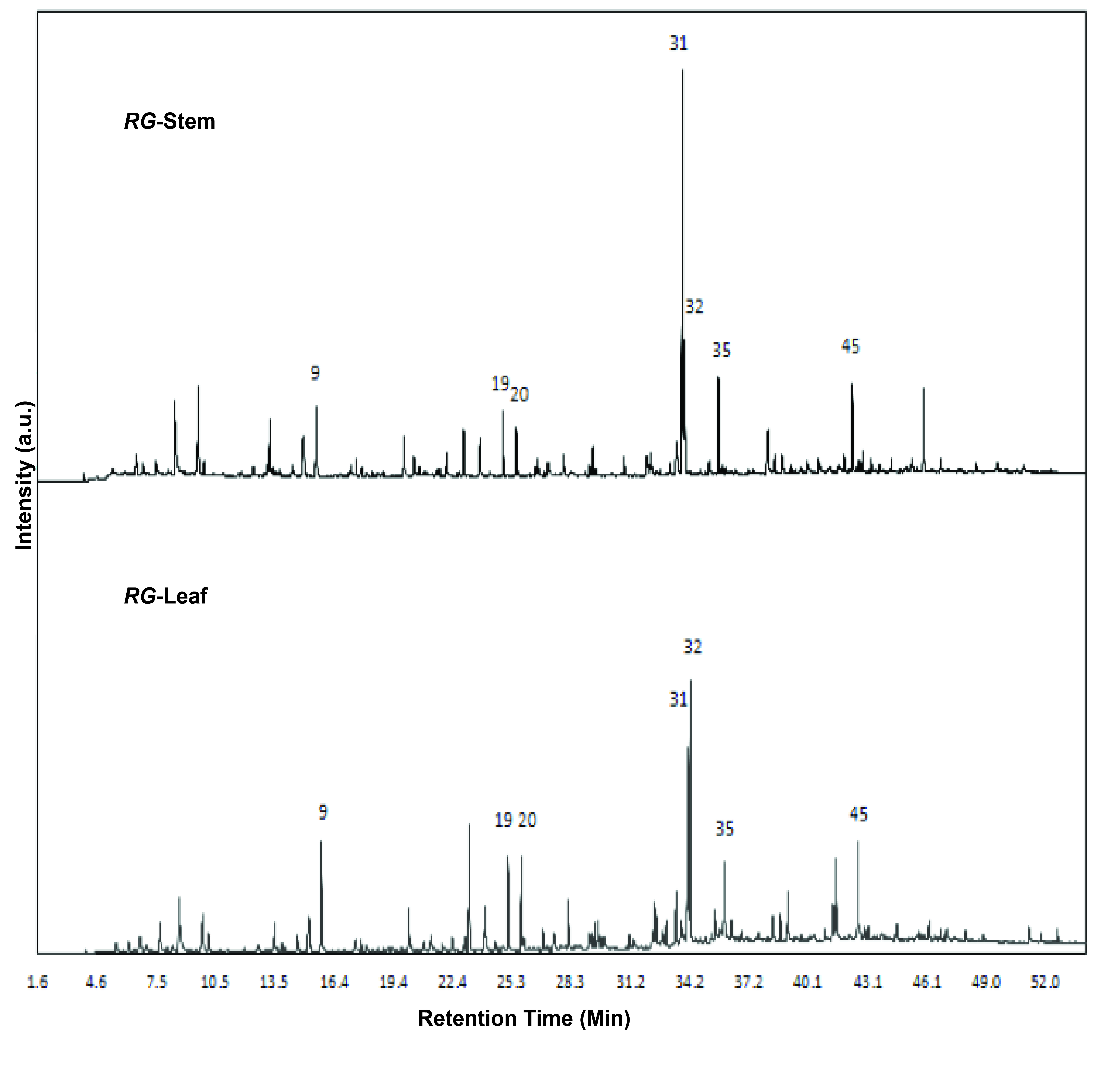

Supplement: Additional file 6: Figure S4 — Representative pyrograms of RG mutant stem and leaf biomass. Numbered peaks on the chromatograms correspond to the peaks reported in Additional file 4: Table S2 and they are typical products seen from pyrolysis of different biomass types. (9) Furfural; (19) phenol; (20) 2-methoxyphenol, (31) 4-vinylphenol; (32) 2-methoxy-4-vinylphenol (35) 2,6-dimethoxyphenol and (45) 4-. [file 1754-6834-6-146-S6.jpeg]
