# Supplementary material for: Sorghum mutant RG displays antithetic leaf shoot lignin accumulation resulting in improved stem saccharification properties
Source: Biotechnol Biofuels. 2013 Oct 9;6:146. doi: 10.1186/1754-6834-6-146 (PMC3852544; doi:10.1186/1754-6834-6-146)
Supplement: Additional file 4: Table S2 — Pryo-GC/MS analysis of RG biomass [Leaf (RGL) and Stem (RGS)]. [file 1754-6834-6-146-S4.docx]

Additional Table 2: Pryo-GC/MS analysis of *RG* biomass [Leaf (RGL) and Stem (RGS)]

|  | **Retention**  **time** | **Compound** | ***RG***  **Leaf** | ***RG* Stem** |
| --- | --- | --- | --- | --- |
| 1 | 6.14 | 2-methylfuran | 0.32 | 0.05 |
| 2 | 6.72 | 2,3-butanedione | 0.76 | 1.16 |
| 3 | 7.70 | hydroxyacetaldehyde | 0.33 | 0.90 |
| 4 | 8.66 | acetic acid | 1.18 | 3.40 |
| 5 | 9.85 | 1-hydroxy-2-propanone | 1.26 | 4.14 |
| 6 | 10.14 | toluene | 0.59 | 0.69 |
| 7 | 13.44 | acetic acid methyl ester | 0.86 | 1.47 |
| 8 | 13.81 | o-xylene | 0.22 | 0.00 |
| 9 | 15.79 | furfural | 2.97 | 2.65 |
| 10 | 17.30 | 2-furanmethanol | 0.00 | 0.66 |
| 11 | 18.01 | 2-methyl-2-cyclopenten-1-one | 0.14 | 0.11 |
| 12 | 18.45 | 2-ethyl-5-methylfuran | 0.00 | 0.02 |
| 13 | 19.00 | 2-cyclopentene-1,4-dione | 0.00 | 0.07 |
| 14 | 20.16 | 1,2-cyclopentanedione | 1.08 | 1.39 |
| 15 | 21.20 | 5-methyl-2-furancarboxaldehyde | 0.27 | 0.02 |
| 16 | 21.83 | 3-methyl-2-cyclopenten-1-one | 0.09 | 0.33 |
| 17 | 22.33 | 2(5H)-furanone | 0.52 | 1.01 |
| 18 | 23.97 | 2-hydroxy-3-methyl-2-cyclopenten-1-one | 1.56 | 2.02 |
| 19 | 25.12 | phenol | 2.19 | 2.10 |
| 20 | 25.79 | 2-methoxyphenol | 2.08 | 1.76 |
| 21 | 26.87 | 2-methylphenol | 0.59 | 0.74 |
| 22 | 27.05 | 3-ethyl-2-hydroxy-2-cyclopenten-1-one | 0.00 | 0.22 |
| 23 | 28.13 | 4-methylphenol | 1.12 | 0.62 |
| 24 | 28.20 | 3-methylphenol | 0.38 | 0.41 |
| 25 | 29.45 | 2-methoxy-4-methylphenol | 0.59 | 0.27 |
| 26 | 29.77 | 2,4-dimethylphenol | 0.27 | 0.11 |
| 27 | 31.18 | 4-ethylphenol | 0.29 | 0.83 |
| 28 | 31.41 | benzoic acid | 0.37 | 0.00 |
| 29 | 32.33 | 4-ethyl-2-methoxyphenol | 1.09 | 1.07 |
| 30 | 33.52 | 1,4:3,6-dianhydro-.alpha.-d-glucopyranose | 1.02 | 0.28 |
| 31 | 34.09 | 4-vinylphenol | 3.44 | 18.59 |
| 32 | 34.23 | 2-methoxy-4-vinylphenol | 5.51 | 6.18 |
| 33 | 34.83 | eugenol | 0.14 | 0.00 |
| 34 | 35.20 | 5-hydroxymethyl-2-furancarboxaldehyde | 1.98 | 0.18 |
| 35 | 35.92 | 2,6-dimethoxyphenol | 1.38 | 3.51 |
| 36 | 36.78 | 2-methoxy-4-(1-propenyl)phenol C | 0.30 | 0.13 |
| 37 | 38.36 | 2-methoxy-4-(1-propenyl)phenol T | 1.98 | 2.52 |
| 38 | 38.74 | 4-methylsyringol | 0.58 | 0.82 |
| 39 | 39.10 | vanillin | 2.62 | 0.67 |
| 40 | 39.30 | 3-hydroxybenzaldehyde | 0.25 | 0.22 |
| 41 | 40.00 | 3-phenyl-2-propenoic acid | 0.44 | 0.00 |
| 42 | 41.51 | 4-Hydroxy-3-methoxyacetophenone | 2.29 | 0.27 |
| 43 | 42.00 | 4-hydroxybenzaldehyde | 1.87 | 0.64 |
| 44 | 42.28 | 3,5-dimethoxyphenol | 0.58 | 0.00 |
| 45 | 42.62 | 4-vinylsyringol | 2.34 | 3.10 |
| 46 | 42.96 | 1-(4-Hydroxy-3-methoxyphenyl)acetone | 0.33 | 0.15 |
| 47 | 43.14 | 2,6-dimethoxy-4-(2-propenyl)phenol | 0.56 | 0.91 |
| 48 | 43.80 | 1-(2-hydroxyphenylethanone) | 2.00 | 0.17 |
| 49 | 44.56 | 2,6-dimethoxy-4-(1-propenyl)phenol C | 0.59 | 0.63 |
| 50 | 46.19 | 2,6-dimethoxy-4-(1-propenyl)phenol T | 1.08 | 3.02 |
| 51 | 47.06 | 4-hydroxy-3,5-dimethoxybenzaldehyde | 0.44 | 0.75 |
| 52 | 48.64 | 4-hydroxy-3,5-dimethoxyacetophenone | 0.57 | 0.75 |
| 53 | 49.07 | 4-((1E)-3-Hydroxy-1-propenyl)-2-methoxyphenol | 0.00 | 0.04 |
| 54 | 49.63 | 4-Hydroxy-2-methoxycinnamaldehyde | 0.20 | 0.32 |
| 55 | 49.70 | 3-(4-hydroxy-3-methoxyphenyl)-2-propenal | 0.00 | 0.13 |
| 56 | 50.70 | 3-(4-hydroxy-3-methoxyphenyl)-2-propenoic acid methyl ester | 0.27 | 0.03 |
|  |  | **Sum Lignin** | **41.54** | **52.24** |
|  |  | **S derivatives** | **7.54** | **13.49** |
|  |  | **G derivatives** | **17.07** | **13.03** |
|  |  | **S/G** | **0.44** | **1.04** |
