# Supplementary material for: Sorghum mutant RG displays antithetic leaf shoot lignin accumulation resulting in improved stem saccharification properties
Source: Biotechnol Biofuels. 2013 Oct 9;6:146. doi: 10.1186/1754-6834-6-146 (PMC3852544; doi:10.1186/1754-6834-6-146)
Supplement: Additional file 7: Table S3 — Ultimate analysis of biomass samples. [file 1754-6834-6-146-S7.docx]

Additional Table 3. Ultimate analysis of biomass samples

| **Biomass** | **C (%)** | **H (%)** | **N (%)** | **O (%)** | **S (ppm)** | **P (ppm)** | **Ash (%)** | **Moisture (%)** | **Calorific Content (KJ/kg)** |
| --- | --- | --- | --- | --- | --- | --- | --- | --- | --- |
| WTL | 43.72 | 6.19 | 2.89 | 39.96 | 2141 | 3699 | 7.15 | 5.83 | 17978 |
| WTS | 42.01 | 6.59 | 0.48 | 48.24 | 475 | 1177 | 2.65 | 10.82 | 16477 |
| *RG*L | 43.58 | 6.07 | 1.04 | 43.74 | 1455 | 2837 | 5.46 | 9.97 | 16784 |
| *RG*S | 41.18 | 6.45 | 1.58 | 45.08 | 988 | 1247 | 5.63 | 8.60 | 16240 |

Biomass assayed were wild type leaf (WTL), stem (WTS); *REDforGREEN* leaf (RGL) and stem (RGS)
