# Supplementary material for: Sorghum mutant RG displays antithetic leaf shoot lignin accumulation resulting in improved stem saccharification properties
Source: Biotechnol Biofuels. 2013 Oct 9;6:146. doi: 10.1186/1754-6834-6-146 (PMC3852544; doi:10.1186/1754-6834-6-146)
Supplement: Additional file 8: Table S4 — List of genes in the phenylpropanoid pathway investigated by RT-PCR. [file 1754-6834-6-146-S8.docx]

| **Gene Name** | **Abbreviation** | **Primer sequence** | **Pathway branch** |
| --- | --- | --- | --- |
| *Actin* | *Act* | ACATTGCCCTGGACTACGAC  TGATGACCTGTCCATCAGGA |  |
| *Cinnamyl alcohol dehydrogenase* | *CAD* | ACCCAAATGTTTCTGGTGCT  GTACGGAGTGTCGGAATCGT | Lignin |
| *Caffeic acid O-methyltransferase* | *COMT* | ACATGCATGCCGGTTTATTT  CAGCGCTTGAGAAATTGTGT | Lignin |
| *Caffeoyl-CoA O-methyltransferase* | *CCoAOMT* | GTGTCATCCGATGTCCGAAT  CTGCCAATCAACAATCATGG | Lignin |
| *Cinnamoyl-CoA reductase* | *CCR* | TGCATGCTTTCCTGATGAGT  ATGGCTGCACCTAATTTTGC | Lignin |
| *4-coumaric acid 3`-hydroxylase* | *C3H1* | CATGGGCACACCACTACAAG  CTTAGCTGAAAGCAGCAGCA | Lignin |
| *Hydroxycinnamoyl transferase* | *HCT* | CCTATCCATGATGCCGACTT  TAGCTTCCGGAACTTCTCCA | Linin |

**Additonal Table 4:** List of genes in the phenylpropanoid pathway investigated by RT-PCR
